# Patient and public involvement in health research in Norway: a survey among researchers and patient organisations

**DOI:** 10.1186/s40900-023-00458-x

**Published:** 2023-07-08

**Authors:** Sigve Nyvik Aas, Marita Borg Distefano, Ingvild Pettersen, Berit Gravrok, Laila Yvonne Nordvoll, Jon Fauskanger Bjaastad, Sameline Grimsgaard

**Affiliations:** 1 Clinical Research Unit, St. Olavs Hospital, Trondheim, Norway; 2 Division of Research and Innovation, Akershus University Hospital, Lørenskog, Norway; 3 Clinical Research Department, University Hospital of North Norway, Tromsø, Norway; 4 Department of Research and Development, Haukeland University Hospital, Bergen, Norway; 5 Division of Psychiatry, Stavanger University Hospital, Stavanger, Norway

**Keywords:** Patient and public involvement, User representatives, Health research

## Abstract

**Background:**

Patient and public involvement (PPI) in health research may improve both the relevance and quality of the research. There is however a lack of research investigating the experiences, attitudes and barriers towards PPI in clinical research in Norway. The Norwegian Clinical Research Infrastructure Network therefore conducted a survey among researchers and PPI contributors aiming to investigate experiences with PPI and identify current challenges for successful involvement.

**Methods:**

Two survey questionnaires were developed and distributed in October and November 2021. The survey targeting 1185 researchers was distributed from the research administrative system in the Regional Health Trusts. The survey targeting PPI contributors was distributed through Norwegian patient organisations, regional and national competence centers.

**Results:**

The response rate was 30% among researchers and was unobtainable from PPI contributors due to the survey distribution strategy. PPI was most frequently used in the planning and conduct of the studies, and less utilized in dissemination and implementation of results. Both researchers and user representatives were generally positive to PPI, and agreed that PPI might be more useful in clinical research than in underpinning research. Researchers and PPI contributors who reported that roles and expectations were clarified in advance, were more likely to experience a common understanding of roles and responsibilities in the research project. Both groups pointed to the importance of earmarked funding for PPI activities. There was a demand for a closer collaboration between researchers and patient organisations to develop accessible tools and effective models for PPI in health research.

**Conclusions:**

Surveys among clinical researchers and PPI contributors indicate overall positive attitudes towards PPI in clinical research. However, more resources, such as budget, time, and accessible tools, are needed. Clarifying roles and expectations, and creating new PPI models under resource constraints can enhance its effectiveness. PPI is underutilized in disseminating and implementing research results, presenting an opportunity for improving healthcare outcomes.

**Supplementary Information:**

The online version contains supplementary material available at 10.1186/s40900-023-00458-x.

## Abstract (plain language summary)

Patient and public involvement (PPI) in health research can make the research more relevant and of better quality. However, in Norway, there has not been much research on the experiences, attitudes, and barriers related to PPI in clinical research. To address this gap, we conducted a survey among researchers and PPI contributors to understand their experiences and identify current challenges. We found that PPI was most common during planning and execution of studies. PPI was less used in the process of sharing the results from the studies, and in the process of putting the findings into practice. Those who reported that roles and expectations were clarified in advance were more likely to have a shared understanding of their roles and responsibilities in the research project. Both groups emphasized the importance of funding for PPI activities. There was also a desire for closer collaboration between researchers and patient organisations to develop accessible tools and for PPI. In summary, the survey revealed a generally positive attitude towards PPI in health research. However, more resources, such as budget, time, and accessible tools, are needed. Clarification of roles and expectations also stand out a crucial part of the PPI process, and should receive much attention in all research projects where PPI is used.

**Supplementary Information:**

The online version contains supplementary material available at 10.1186/s40900-023-00458-x.

## Background

The focus on user involvement in health research has increased both internationally and in Norway over the past years [1, 2]. User involvement, often referred to as patient and public involvement (PPI), reflects the notion that research should be carried out “with” or “by” members of the public rather than “to”, “about” or “for” them [3]. By involving patients and the public in the research process, PPI has the potential to improve the quality, relevance, impact and implementation of health research [4, 5]. PPI may also empower the users of health and social care services and thus contribute to a democratization of the research process [2].

Attitudes, approaches, and experiences with PPI vary considerably among countries [6]. A large body of the literature about PPI originates from the United Kingdom (UK), where PPI is well established [6]. A cross-sectional analysis of health research published in BMJ open in 2000 showed that inclusion of PPI varied considerably in research by location (country of origin), by the applied research methodology, by health topic and funding source [6]. These differences most likely reflect difference in research traditions as well as national socio-political events and trends.

Norway has applied a “top-down” strategy to implement PPI [7, 8]. The Norwegian national action plan for clinical studies states that PPI is essential to improve relevance and quality of health research [9]. Since 2016, consideration of PPI has been mandatory in all health research funded by the Norwegian Regional Health Authorities (RHA) [10, 11], which is the main funding body for clinical research in Norway. Both national and international guidance emphasize that PPI should be considered in all phases of the research process, from deciding on research questions, to planning, designing and conducting the study, and finally in the dissemination and implementation of research results. In summary, PPI is becoming increasingly important and may thus require new collaborative structures and tools for researchers, patients and the public.

There is a lack of research investigating public/patient representatives´ and researchers’ attitudes toward PPI in research in Norway and across Europe [1]. The Norwegian university hospitals’ infrastructure partnership for support of clinical research, NorCRIN [12], therefore conducted a survey to collect information from researchers and patient organisations on their attitudes to, and experiences with PPI in the research process. The aim of the study was to identify current challenges and barriers for the successful utilization of PPI in health research.

## Methods

### Study design and data collection

The current study was conducted by the Norwegian Clinical Research Infrastructure Network (NorCRIN), which is the Norwegian university hospitals’ infrastructure partnership [12] aiming to support clinical research. Two online surveys were developed, one for researchers, and one for PPI contributors. The two surveys contained some common questions, allowing for cross-sectional comparisons between researchers and PPI contributors. Pilot surveys were sent to a small group of researchers and patient representatives to obtain feedback on questions and forms as part of the process of developing the final survey. The survey to PPI contributors was reviewed by two user representatives. The user representatives had their background from patient and user organisations and were representatives with the perspectives from both users and next of kin. The aim of involving them in the survey development was to ensure that all relevant questions were addressed, and that questions were asked in a clear and concise manner. The user representatives had good knowledge of Norwegian patient organisations and contributed valuable information about relevant channels for the distribution of the survey. They also wrote the cover letter that was submitted together with the survey. The surveys were designed so that follow-up questions were given if the respondent reported experience with PPI in research. The survey targeting PPI contributors had a minimum of five and a maximum of 14 questions, depending on whether the respondent had PPI experience. Altogether 11 questions were closed-ended questions with alternatives. Four questions also contained a field for free-text comments. The survey additionally contained three open-ended questions without alternatives. The survey is presented in its entirety in Additional file 1: Appendix A.

The survey targeting researchers had a minimum of 9 and a maximum of 16 questions, depending on the level of PPI experience. Altogether 12 questions were closed-ended questions with alternatives. Two questions had a field for free-text comments, and the survey also contained three open-ended questions (Additional file 1: Appendix B). All researchers were also asked to specify their research activity, according to the Health Research Classification System [13].

Both surveys were distributed in October and November 2021. Researcher’s e-mail addresses were obtained through eRapport [14], which is the administrative register for all research projects funded by the RHA. The researchers received one e-mail reminder. The survey to PPI contributors was sent to a comprehensive list of patient organisations, as well as national and regional competence centres. Some were umbrella organisations (e.g., The Norwegian Federation of Organisations of Disabled People) and were asked to forward the survey to their member organisations. All recipients were encouraged to forward the survey to all employees and members. Two reminder e-mails were sent to the PPI-contributors. The surveys were closed on November 30^th^, 2021. Both surveys were answered anonymously, and the data collected did not allow for backward identifications of respondents.

We used Research Electronic Data Capture (RedCAP) to collect the survey data. The study was not mandatory for submission to the Regional Committee for Medical and Health Research Ethics because the survey questions did not cover health data. Data were collected anonymously, and the study was approved by the privacy officer at the University Hospital of North Norway.

### Data analysis

The data was analyzed using SPSS. We used descriptive analyses (proportions and percentages) to present study results and Pearson Chi-Square to test between-group differences. Two-sided *p*-values < 0.05 were considered statistically significant. Figures were prepared in GraphPad Prism. Only results for the closed-ended questions are presented in the present article. No missing data were imputed.

The user representatives were invited to a workshop to discuss study results, and thus contributed to the interpretation of the results. A GRIPP2 checklist is provided in Additional file 1: Appendix H.

## Results

The researcher survey was sent to 1185 researchers who received research funding from the Regional Health Authorities in 2021. Altogether 355 (30%) researchers completed the survey. The patient organisation survey was sent to 303 addresses and completed by 595 individuals. The patient organisations were encouraged to forward the survey to their members, and due to this distribution strategy, we do not have information about how many received the survey and the corresponding response rate.

Most results presented are based on the proportion of respondents who reported experience with PPI. Altogether 314 (88.4%) researchers reported PPI-experience. Altogether 129 (21.7%) of 595 respondents from the patient organisation sample reported PPI-experience. This subgroup is referred to as “PPI contributors”. Not all respondents answered all questions. Thus, the number of respondents vary slightly between questions.

### Roles and expectations

**Fig. 1:**
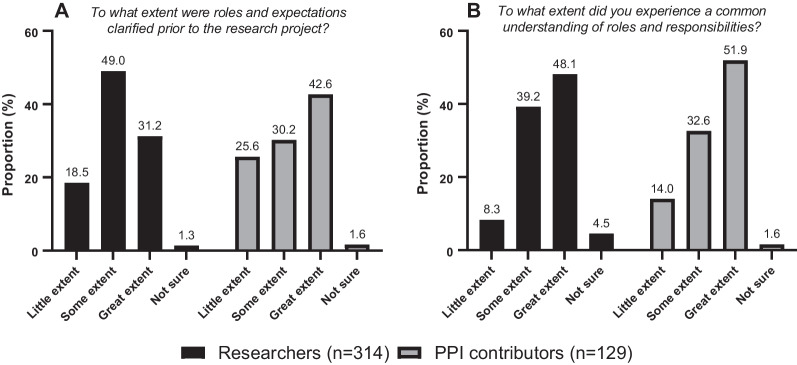
**A** display to what extent roles and expectations were clarified prior to initiation of the research projects.**B** display to what extent researchers and PPI contributors experienced a common understanding of roles and responsibilities (retrospective assessment)

Both researchers with PPI experience and PPI contributors were asked to what extent a clarification of roles and expectations was conducted prior to initiation of the research project (Fig. 1A). They were also asked to what extent they *experienced* a common understanding of roles and responsibilities in the project in which their experience resided (retrospective assessment) (Fig. 1B).

Respondents reporting that roles and expectations to a great extent were clarified prior to the research project were significantly more likely to experience a common understanding of roles and responsibilities when looking back at the project (*p* < 0.001 for researchers and PPI contributors, Fig. 1A, B). See Additional file 1: Appendix C for details.

### Timing and nature of involvement

**Fig. 2:**
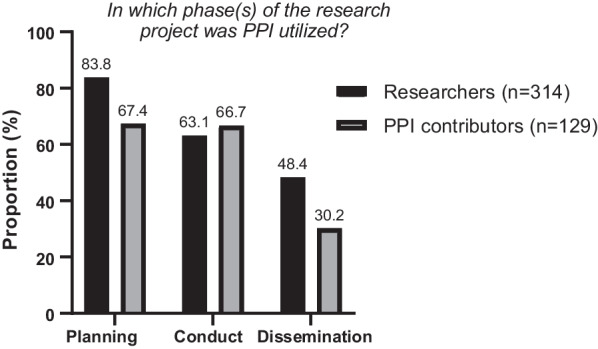
Stage of the research project in which PPI was utilized, according to researchers and PPI contributors. Selecting more than one alternative was possible

Figure 2 shows that patient and public involvement was most frequent in the early planning and during study conduct. Only one in three PPI contributors had been involved in dissemination of study results.

Respondents were asked more specifically about *how* the user had been involved. The respondents could select from a list of nine alternatives (presented in Additional file 1: Appendix D). The PPI contributors reported that they had given input to the research question (63%) and supplied background information to the project (45%). A lower proportion of PPI contributors reported involvement in discussion/interpretation, dissemination, and implementation of the research results (6–30%). In general, researchers agreed with the PPI contributors on this topic.

### Economy

**Fig. 3:**
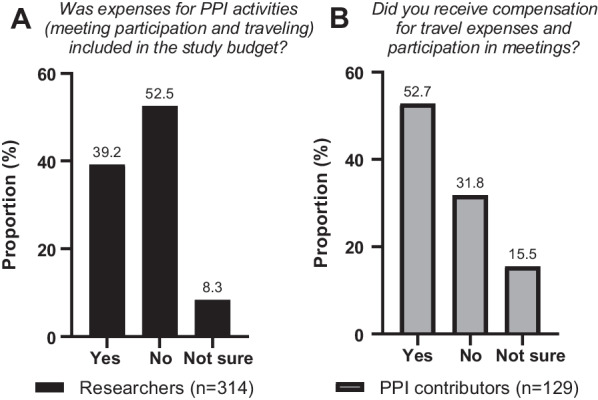
Budgeting and compensation of PPI activities, according to researchers (**A**) and PPI contributors (**B**)

Figure 3A shows that the majority of the researchers reported that expenses for PPI were *not* included in the study budget. Approximately one-third of PPI contributors reported that they did *not* receive compensation for their involvement (Fig. 3B), and a similar proportion reported that their involvement in the research project was more time consuming than expected (Additional file 1: Appendix E).

### Quality and relevance of research

All respondents were asked whether they considered that patient and public involvement can improve the quality and relevance of research. All respondents were also asked whether patient involvement may identify needs and challenges for patients and relatives. Results are presented below (Table 1).

**Table 1:** Attitude towards PPI in health research

*Question A: Can PPI improve the quality and/or relevance of research?*	Yes (%)	No (%)	Not sure (%)
Researchers (n: 355)	71.3	10.4	18.3
Patient organisation respondents (n: 592)	96.3	0.7	3
*Question B: Can PPI help identify needs and challenges for patients and relatives?*	Yes (%)	No (%)	Not sure (%)
Researchers (n: 355)	84.2	4.8	11
Patient organisation respondents (n: 595)	96.6	1	2.4

Respondents from patient organisations were more likely to respond that PPI can improve the quality and relevance of research as compared to researchers (*p* < 0.001, Table 1). Respondents from patient organisations were also more likely to respond that PPI can identify needs and challenges for patients and relatives as compared to researchers (*p* < 0.001, Table 1). Altogether, 77% of researchers with direct PPI-experience responded that “PPI can improve the quality and/or relevance of research”, as compared to 38% of researchers without such experience (*p* < 0.001). The answers to this question differed across the HRCS research areas/categories. 65% of researchers in the category *underpinning research* answered “yes” to the question whether PPI can improve the quality and relevance of research, as compared to 84% and 87% of researchers in the categories *health and social care services research* and *management of diseases and conditions*, respectively. Complete results are presented in Additional file 1: Appendix F.

### Measures to improve patient and public involvement in research

**Fig. 4:**
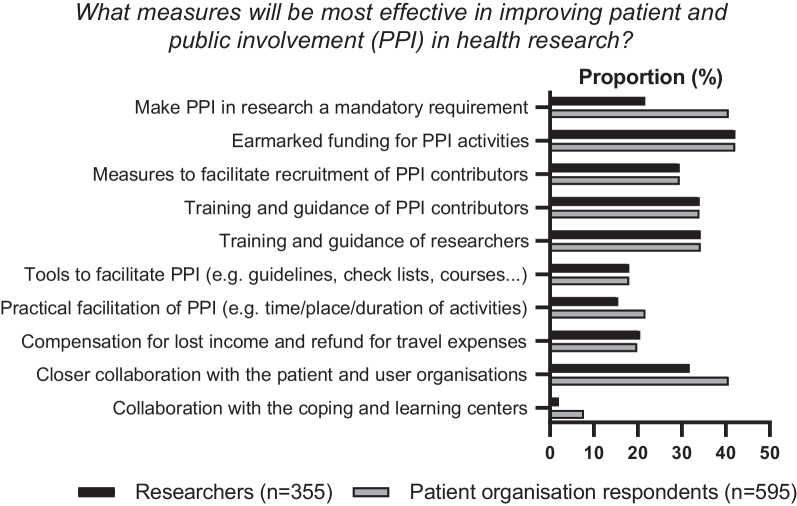
The surveys suggested ten different measures/initiatives to improve PPI in research, and all respondents were asked to select up to three measures from the list**.** The figure displays the proportion (%) of researchers and patient organisation respondents selecting the various measures

All respondents, regardless of PPI experience, were asked about their opinions about what measures would be most effective in improving patient and public involvement in research. The level of agreement between researchers and respondents from patient organisations is shown in Fig. 4. Both groups pointed to the importance of earmarked funding for PPI activities, closer collaboration between researchers and patient organisations, and measures to facilitate recruitment of user representatives to the research projects. Among researchers, *training and guidance of researchers* was the second most selected measure. The two groups differed in their responses to the statement “PPI should be mandatory in health research”; 40% of patient organisations respondents agreed, compared to 22% of researchers (*p* < 0.001 for difference between groups).

## Discussion

To our knowledge, this is the largest survey to address experiences with patient and public involvement in Norwegian health research, that also allowed us to compare experiences of researchers and PPI contributors. Researchers with PPI experience and PPI contributors were generally positive and agreed that PPI may contribute to improve both the quality and relevance of health research. The survey results also showed areas for improvement, as well as pinpointing infrastructure and competence needs for both PPI contributors and researchers. Some challenges, such as questions of payment and compensation, training and availability of representatives, show the need for a well-organized system to facilitate user involvement in research. The survey provides an overview of current challenges and barriers and may thus have implications for the development and implementation of measures to improve user involvement in Norwegian clinical research.

### Challenges in PPI: roles and expectations, and timing of involvement

Previous research show that a clarification of roles and expectations is crucial for successful PPI in research [15–17]. A systematic review from 2014 showed that challenges were more frequently reported in studies where service users were involved sporadically in the study and with no clear role [4]. In line with this, our results show that both researchers and PPI contributors were significantly more likely to report a great extent of common understanding of roles and responsibilities, if such roles and responsibilities were clarified prior to the research project. Developing tools to facilitate such a clarification between researchers and PPI contributors may be useful.

A research project can be divided into three main phases: study planning, study conduct, and finally analysis, dissemination and implementation of results. Our results show that PPI is most frequently used in the planning and conduct of the studies, and less utilized in dissemination and implementation of results. This finding is also in line with previous research, where engagement with PPI contributors regarding the dissemination of research findings was generally found to be lacking [18]. Dissemination of research results are vital steps in the research process where PPI can contribute to public focus on research results, the development of guidelines, and information on how research results can be used in clinical practice. PPI can thus improve services and outcomes, contribute to reduce research waste, and provide the society with payback for its research investment. Increasing PPI activities in the later phases of a research project stands out as “low-hanging fruit” to facilitate implementation of research results to clinical practice.

Although our survey indicate that PPI is mostly utilized in the planning phase of the studies, many PPI contributors reported that they were included too late in the process. Some funding schemes request PPI in grant applications for projects to be funded. This is an incentive to early PPI and may increase the likelihood of actual influence.

Taken together, our results show that both clarification of roles and timing of involvement are important factors for meaningful PPI. These experiences were already put forward by Brett and colleagues in 2014, when they reported that a clarification of roles and expectations, as well as involvement throughout the study, would increase the positive impact of PPI [4]. This underscores that there is general agreement that clarification of roles and timing of involvement is important, and there is room for improvement on how to solve these issues. Our results show that after many years of focus on PPI, there is still a need for more concrete measures to ensure that both clarification of roles and involvement throughout the different phases of research are taken into account.

We propose that during the initiation of research projects, researchers and PPI contributors discuss and agree on the timing of user involvement, their role throughout different project phases, and the allocation of resources dedicated to PPI. Additionally, we recommend the development of user-friendly tools and checklists to facilitate effective communication and agreement between researchers and PPI contributors in clinical research projects.

### Resource constraints are barriers to successful involvement

The survey highlighted several barriers to PPI in health research. The results indicate that many health research projects lack funding for PPI activities, and both researchers and PPI contributors pointed to earmarked funding as a key measure to strengthen user involvement. A clinical trials unit at Swansea University has developed a standard operating procedure to guide researchers on this topic [19]. They propose allocation of 1% of the total research budget as a minimum resource to involve service users and allow enough time to facilitate active involvement. A similar approach can be valuable also in the Nordic countries. The budgets should go beyond mere participation in meetings and encompass other essential PPI activities, such as providing feedback on the study protocol, disseminating written information to research subjects, and creating questionnaires. Additionally, it is crucial to establish funding schemes and PPI models that enable early project involvement even before funding applications are submitted.

Lack of time is another barrier to successful PPI, and this was also evident from our survey. It is important to initiate measures to make the PPI process as efficient and seamless as possible. The use of electronic platforms for communication may reduce time, cost, and impact on the environment. In summary, resource constraints are prominent barriers to successful PPI. We need to develop new and collaborative models for PPI, taking into account that PPI contributors are a limited resource that must be used wisely. Consulting and discussion with the patient organisations and user representatives will aid the development of appropriate models and arenas for collaboration and involvement.

Our survey also points to lack of competence as a potential barrier to successful PPI in health research. A broad range of resources and training material have already been developed, including e-learning courses, guideline documents and various checklists [20–23], both at international and national levels. PPI can easily be improved by making researchers familiar with already existing tools and resources. Patient organisations, funding bodies and research networks can engage and collaborate to collect, develop and present resources, models, and tools for PPI.

Other studies have pointed out challenges to successful PPI that are more likely to emerge from interview data, such as difficulties in balancing academic requirements versus user-perspectives, tokenistic user involvement and power-imbalance, as well as difficulties in recruiting representative users to research projects [4, 8, 24]. Nonetheless, recent reviews show that the importance and value of PPI are increasingly recognized [1], and the development and implementation of measures to improve user involvement in clinical research will be an important step forward.

### Is the usefulness of PPI dependent on the research activity?

Researchers working with patient-centred research were generally more positive to PPI as compared to researchers working in underpinning/etiology research. It is challenging to implement meaningful PPI in underpinning and etiology (preclinical) research [19]. “Users” of preclinical research are often researchers in other fields and may contribute meaningfully as “users representatives” in these research projects. “Patient” user representatives are limited resources and should be used in studies where their involvement has impact, and where PPI is likely to improve the quality and relevance of the project.

### Impact of user involvement in the current study

The user representatives participated to develop the survey by giving substantial input to the survey topics. They contributed to phrasing of the survey questions, which led to more relevant and precise questions. They identified appropriate distribution channels, and to address the respondents in the cover letter submitted together with the survey. It is likely that their involvement also had an impact on the number of respondents, in particular from patient organisations. They contributed to discussion and interpretation of the survey results in a workshop with the researchers, and they have also been involved in dissemination of preliminary results. We experienced that user involvement contributed to all phases of the research project and improved both the relevance and quality of the present study.

### Limitations and strengths

Surveys have limitations when it comes to generalizability. The sample size, participation and composition of respondents play a significant role in determining how well survey results can be applied to a larger population. The survey addressing researchers was sent to those who had funding from the Regional Health Authorities in 2021, i.e., the main funding body for clinical research in Norway. Researchers with PPI experience were therefore overrepresented among the respondents and the survey provides limited information from researchers without PPI experience. The response rate among researchers was modest (30%), and the results should be interpreted with caution. Due to the distribution strategy, the response rate in the patient organisation sample is unknown, and this is a limitation. Moreover, by utilizing a binary yes/no format for many questions instead of a Likert scale, we may have overlooked important subtleties and variations in participants' responses.

The strengths of our study are nevertheless the large number of respondents among clinical researchers and patient organisations. The two samples reflect a broad range of roles, perceptions, and experiences with PPI in Norway.

## Conclusions

The surveys conducted among clinical researchers and PPI contributors showed that both groups are generally positive to PPI in clinical research. There is however a need for more resources to PPI - e.g. allocated budget, dedicated time, and tailored and accessible tools. Moreover, a thorough clarification of roles and expectations stand out a crucial part of the PPI process, and should receive much attention in all research where PPI is utilized. Finally, PPI is less used in the dissemination and implementation of research results, and this is an area where PPI can facilitate and contribute to improve health care.

### Supplementary Information

**Additional file 1**. Surveys, supplementary figures, and GRIPP2 short form.

## Data Availability

The datasets generated in the study are available from the corresponding author on reasonable request.

## Abbreviations

HRCS: Health Research Classification System
NorCRIN: Norwegian Clinical Research Infrastructure Network
PPI: Patient and public involvement
RedCAP: Research electronic data capture
RHA: Regional health authorities
SOP: Standard operating procedure
UK: United Kingdom

## References

[1] Biddle MSY, Gibson A, Evans D (2021). Attitudes and approaches to patient and public involvement across Europe: a systematic review. Health Soc Care Commun.

[2] Shippee ND, Domecq Garces JP, Prutsky Lopez GJ, Wang Z, Elraiyah TA, Nabhan M (2015). Patient and service user engagement in research: a systematic review and synthesized framework. Health Expect.

[3] Caress AL, Ford A, Roberts L, Turner K, Ward D, Williamson T. Briefing notes for researchers: public involvement in NHS, public health and social care research. 2012;1-49. https://www.invo.org.uk/wp-content/uploads/2012/04/INVOLVEBriefingNotesApr2012.pdf. Accessed 15 June 2023.

[4] Brett J, Staniszewska S, Simera I, Seers K, Mockford C, Goodlad S (2017). Reaching consensus on reporting patient and public involvement (PPI) in research: methods and lessons learned from the development of reporting guidelines. BMJ Open.

[5] Petit-Zeman S, Locock L (2013). Health care: bring on the evidence. Nature.

[6] Lang I, King A, Jenkins G, Boddy K, Khan Z, Liabo K (2022). How common is patient and public involvement (PPI)? Cross-sectional analysis of frequency of PPI reporting in health research papers and associations with methods, funding sources and other factors. BMJ Open.

[7] Ministry of Health and Care Services, Norway. HelseOmsorg21. Et kunnskapssystem for bedre folkehelse. Nasjonal forsknings- og innovasjonsstrategi for helse og omsorg. https://www.helseomsorg21.no/. Accessed 15 June 2023.

[8] Blix BH, Hamran T. Brukermedvirkning og representasjon i helse- og omsorgsforskning. Tidsskrift for omsorgsforskning. 2021;7(3):1–15.

[9] Ministry of Health and Care Services, Norway. The Norwegian national action plan for clinical studies. 2021–2025. https://www.regjeringen.no/contentassets/c3dcdb95b7d741319c62642865afadad/i-1206b_kliniske_studier_uu.pdf. Accessed 15 June 2023.

[10] Helse Vest, Helse Nord, Helse Midt-Norge, Helse Sør-Øst. Veileder for brukermedvirkning i helseforskning i spesialisthelsetjenesten. 2018.

[11] Kasper J, Anne Regine Lager AE, Rumpsfeld M, Kienlin S, Hoel Smestad K, Bråthen T et al. Status report from Norway: Implementation of patient involvement in Norwegian health care. Zeitschrift für Evidenz, Fortbildung und Qualität im Gesundheitswesen. 2017; 123–124: 75–80 10.1016/j.zefq.2017.05.015 28546052

[12] Norwegian national research infrastructure body (NorCRIN). https://www.norcrin.no/. Accessed 15 June 2023.

[13] Health Research Classification System (HRCS), UK Clinical Research Collaboration (UKCRC). https://hrcsonline.net/research-activities/. Accessed 15 June 2023.

[14] eRapport - register for research projects. Regionalt kompetansesenter for klinisk forskning. https://forskningsprosjekter.ihelse.net/. Accessed 15 June 2023

[15] Harrison JD, Auerbach AD, Anderson W, Fagan M, Carnie M, Hanson C (2019). Patient stakeholder engagement in research: a narrative review to describe foundational principles and best practice activities. Health Expect.

[16] Gray-Burrows KA, Willis TA, Foy R, Rathfelder M, Bland P, Chin A (2018). Role of patient and public involvement in implementation research: a consensus study. BMJ Qual Saf.

[17] Mathie E, Wythe H, Munday D, Millac P, Rhodes G, Roberts N (2018). Reciprocal relationships and the importance of feedback in patient and public involvement: a mixed methods study. Health Expect.

[18] Blackburn S, McLachlan S, Jowett S, Kinghorn P, Gill P, Higginbottom A (2018). The extent, quality and impact of patient and public involvement in primary care research: a mixed methods study. Res Involv Engagem.

[19] Maccarthy J, Guerin S, Wilson AG, Dorris ER (2019). Facilitating public and patient involvement in basic and preclinical health research. PLoS One.

[20] Evans BA, Bedson E, Bell P, Hutchings H, Lowes L, Rea D, West Wales Organisation for Rigorous Trials in Health (WWORTH) (2013). Involving service users in trials: developing a standard operating procedure. Trials.

[21] Bagley HJ, Short H, Harman NL, Hickey HR, Gamble CL, Woolfall K (2016). A patient and public involvement (PPI) toolkit for meaningful and flexible involvement in clinical trials - a work in progress. Res Involv Engagem.

[22] Staniszewska S, Brett J, Simera I, Seers K, Mockford C, Goodlad S (2017). GRIPP2 reporting checklists: tools to improve reporting of patient and public involvement in research. BMJ.

[23] Smits DW, van Meeteren K, Klem M, Alsem M, Ketelaar M (2020). Designing a tool to support patient and public involvement in research projects: the involvement matrix. Res Involv Engagem.

[24] Tritter JQ (2009). Revolution or evolution: the challenges of conceptualizing patient and public involvement in a consumerist world. Health Expect.

