# Supplementary material for: Patient and public involvement in health research in Norway: a survey among researchers and patient organisations
Source: Res Involv Engagem. 2023 Jul 8;9:48. doi: 10.1186/s40900-023-00458-x (PMC10329785; doi:10.1186/s40900-023-00458-x)
Supplement: Supplementary file 1 — Additional file 1. Surveys, supplementary figures, and GRIPP2 short form. [file 40900_2023_458_MOESM1_ESM.docx]

**Supplementary material**

**Appendix A –** Patient Organisation Survey

**Question 1**

Do you have experience with patient and public involvement in research?

- Yes
- No

If Yes:

- Yes, direct experience (from specific research projects)
- Yes, indirect experience (e.g. allocation of research funds)

Text:

The next questions are related to your experiences with patient and public involvement in research. If you have experience from more than one project, we ask you to consider the last of these.

**Question 2** (only if Yes on Question 1)

If yes, how were you recruited as user representative?

- Through patient- or user organization
- General Practitioner (GP) or nurse/public health nurse
- At the hospital / in connection with treatment
- Was contacted directly by the practitioner/researcher
- Other (specify)

**Question 3** (only if Yes on Question 1)

To what extent did you discuss and clarify your role, tasks and expected time comitted as a user representative at the start of the research project?

- Little extent
- Some extent
- Great extent
- Not sure

**Question 4** (only if Yes on Question 1)

To what extent did you experience that the PPI contributor(s) and researcher(s) had a common understanding of roles and responsibilities in the research project?

- Little extent
- Some extent
- Great extent
- Not sure

**Question 5** (only if Yes on Question 1)

Did you receive a refund for your travel expenses and compensation for your participation in meetings?

- Yes
- No
- Not sure

**Question 6** (only if Yes on Question 1)

In which phase of the research project where you involved as a user representative/PPI? (see figure, multiple answers possible).

- Planning
- Conduct
- Dissemination

**Question 7** (only if Yes on Question 1)

How did you as a PPI contribute to the research project? (multiple answers possible)

- Had ideas and inputs to the research question/topic in the project
- Supplying background information for the project
- Assisting in fund applications
- Developing information for study participants
- Recruitment of research subjects
- Practical assistance in the conduct of the project
- Discussion and interpretation of results
- Dissemination of research findings
- Implementation of research findings
- Other (specify)

**Question 8 a** (only if Yes on Question 1)

Do the following quotes describe your experience as a PPI contributor in research?

- I was involved early in the research project (Yes – No – Not sure)
- I was involved in formulating research questions (Yes – No – Not sure)
- I suggested research questions to be investigated in the project (Yes – No – Not sure)
- I experienced being listened to (Yes – No – Not sure)
- I experienced having actual influence (Yes – No – Not sure)
- My competence on PPI in research was adequate (Yes – No – Not sure)
- I was comfortable in the role as PPI contributor (Yes – No – Not sure)
- It was more time consuming that I expected (Yes – No – Not sure)
- I was treated like an equal contributor in the research project (Yes – No – Not sure)
- I was involved in processes and meetings that I found important (Yes – No – Not sure)

**Question 8 b** (only if Yes on Question 1)

Do you have other experiences?

(free text field)

**Question 9** (only if Yes on Question 1)

Do you have a specific example of successful PPI in research?

(free text field)

**Question 10** (only if Yes on Question 1)

Do you have a specific example of less successful PPI in research?

(free text field)

**Question 11**

Which of these measures do you think will be most effective in terms of strengthening PPI in research in Norway? (select up to three measures).

- Mandatory requirement of PPI in research
- Earmarked funding for PPI activities
- Measures to facilitate recruitment of PPI contributors
- Training and guidance of PPI contributors
- Training and guidance of researchers
- Tools to facilitate PPI (e.g. guidelines, check lists, courses..)
- Practical facilitation/arrangements of PPI (e.g. time/place/duration of activities)
- Compensation for lost income and refund for travel expenses
- Closer collaboration with the patient and user organisations
- Collaboration with the Learning and Mastery Services centers in Health
- Other suggestions (specify)

Specify other suggestions

(free text field)

**Question 12**

Do you think PPI can contribute to better research? (free text field)

**Question 13**

Do you think PPI can identify important challenges for patients and next of kin? (free text field)

**Question 14**Do you have other comments related to PPI in research, that have not been covered by the questions above? (free text field)

**Appendix B** – Researcher survey

**Question 1**

In which health region are you employed?

- Helse Nord
- Helse Midt
- Helse Vest
- Helse Sør-Øst

**Question 2**

Do you have experience with PPI in research?

- Yes
- No

If Yes:

- Yes, direct experience from specific research projects
- Yes, indirect experience (e.g. allocation of research funds)

**Question 3** (only if No on Question 2)

What is the reason that you do not have experience with PPI in your research? (selection of multiple alternatives possible)

- It has never been a topic
- No dedicated time for it
- Lack of funding
- Not relevant for my research field
- Not sure how to do it
- Lack of PPI contributors
- Other (specify)

**Text:**

The next questions are related to your experiences with patient and public involvement in research. If you have experience from more than one project, we ask you to consider your latest experience.

**Question 4** (only if Yes on Question 2)

To what extent did you discuss and clarify the role, tasks and expected use of time with the PPI contributor at the start of the research project?

- Little extent
- Some extent
- Great extent
- Not sure

**Question 5** (only if Yes on Question 2)

To what extent did you experience that the PPI contributor(s) and researcher(s) had a common understanding of roles and responsibilities in the research project?

- Little extent
- Some extent
- Great extent
- Not sure

**Question 6** (only if Yes on Question 2)

In what phase of the research project was the PPI contributor involved? (selection of multiple alternatives possible)

- Planning
- Conduct
- Dissemination

**Question 7** (only if Yes on Question 2)

How was the PPI contributor involved in the project(s)? (selection of multiple alternatives possible)

- Had ideas and inputs to the issues/research question/topic in the project
- Supplying background information for the project
- Assisting with fund applications
- Developing the information for study participants
- Recruitment of research subjects
- Practical assistance in the conduct of the project
- Discussion and interpretation of results
- Dissemination of research findings
- Implementation of research findings

**Question 8** (only if Yes on Question 2)

Were expenses for PPI activities (meeting participation and traveling) included in the study budget?

- Yes
- No
- Not sure

**Question 9** (only if Yes on Question 2)

Do you have a specific example of successful PPI in research?

(free text field)

**Question 10** (only if Yes on Question 2)

Do you have a specific example of less successful PPI in research?

(free text field)

**Question 11**

Which of these measures do you think will be most effective in terms of strengthening user involvement in research in Norway? (select up to three measures).

- Mandatory requirement of PPI in research
- Earmarked funding for PPI activities
- Measures to facilitate recruitment of PPI contributors
- Training and guidance of PPI contributors
- Training and guidance of researchers
- Tools to facilitate PPI (e.g. guidelines, check lists, courses..)
- Practical facilitation/arrangements of PPI (e.g. time/place/duration of activities)
- Compensation for lost income and refund for travel expenses
- Closer collaboration with the patient and user organisations
- Collaboration with the Learning and Mastery Services centers in Health
- Other suggestions (specify)

**Question 12**

To what extent do you agree with the following statement: “My competence on the topic of PPI is adequate”.

- Completely agree
- Partly agree
- Partly disagree
- Completely disagree
- Not sure

**Question 13**

Do you think PPI can improve the quality and/or relevance of research?

**Question 14**

Do you think PPI can identify important needs and challenges for patients and next of kin?

**Question 15**

Do you have other comments related to PPI in research, that have not been covered by the questions above?

**Question 16**

Which research categories are most descriptive of your research ( HRCS Hurtigveieldning forskningsaktivitet og helsekategorier v3.xlsx (helseomsorg21monitor.no)? (selection of multiple alternatives possible)

- Underpinning research
- Aetiology
- Prevention of disease and conditions
- Detection screening and diagnosis
- Development of treatments and therapeutic interventions
- Evaluation of treatments and therapeutic interventions
- Management of diseases and conditions
- Health and social care services research

**Appendix C** – Roles, responsibilities, and expectations (proportions in percentage)


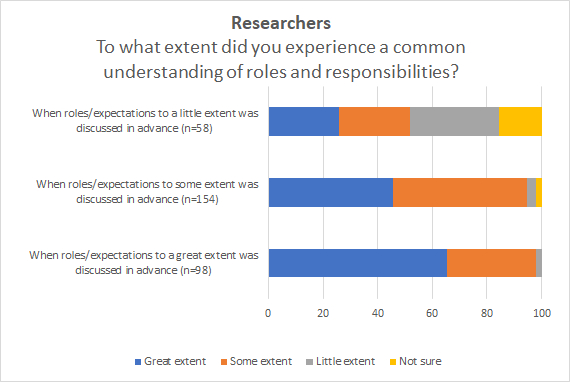


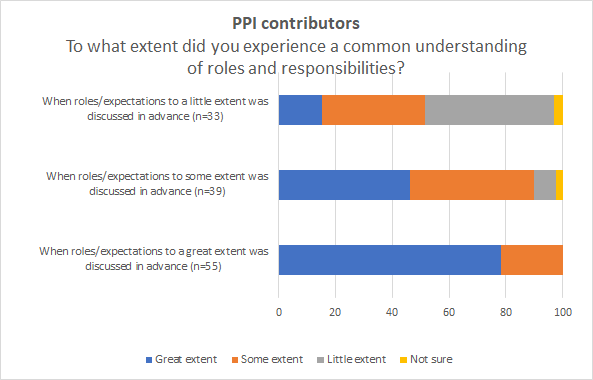


**Appendix D** – Nature of patient and public involvement (proportions in percentage)

**Appendix E** – Experiences among the PPI contributors

**Appendix F** – Research category and attitude towards patient and public involvement

**Appendix G** – PPI competence among researchers

**APPENDIX H** – GRIPP2 short form

| Section and topic | Item | Reported on page No |
| --- | --- | --- |
| Aim | Report the aim of PPI in the study | Page 5 |
| Methods | Provide a clear description of the methods used for PPI in the study | Page 5, 7 |
| Study results | Outcomes—Report the results of PPI in the study, including both positive and negative outcomes | Page 16 |
| Discussion and Outcomes | Comment on the extent to which PPI influenced the study conclusions overall. Describe positive and negative effects | Page 16 |
| Reflections/critical perspective | Comment critically on the study, reflecting on the things that went well and those that did not, so others can learn from this experience | Page 16 |

PPI patient and public involvement
